# Reservoir Dam Surface Deformation Monitoring by Differential GB-InSAR Based on Image Subsets

**DOI:** 10.3390/s20020396

**Published:** 2020-01-10

**Authors:** Peng Wang, Cheng Xing, Xiandong Pan

**Affiliations:** 1 School of Environmental Science and Engineering, Suzhou University of Science and Technology, No. 99, Xuefu Road, Huqiu District, Suzhou 215009, China; 2 School of Geodesy and Geomatics, Wuhan University, No. 129, Luoyu Road, Hongshan District, Wuhan 430079, China; chxing@sgg.whu.edu.cn (C.X.); xdpan@whu.edu.cn (X.P.)

**Keywords:** ground-based synthetic aperture radar (GB-SAR), reservoir dam, image subset, differential interferometry, deformation monitoring

## Abstract

Ground-based synthetic aperture radar interferometry (GB-InSAR) enables the continuous monitoring of areal deformation and can thus provide near-real-time control of the overall deformation state of dam surfaces. In the continuous small-scale deformation monitoring of a reservoir dam structure by GB-InSAR, the ground-based synthetic aperture radar (GB-SAR) image acquisition may be interrupted by multiple interfering factors, such as severe changes in the meteorological conditions of the monitoring area and radar equipment failures. As a result, the observed phases before and after the interruption cannot be directly connected, and the original spatiotemporal datum for the deformation measurement is lost, making the follow-up monitoring results unreliable. In this study, a multi-threshold strategy was first adopted to select coherent point targets (CPTs) by using successive GB-SAR image sequences. Then, we developed differential GB-InSAR with image subsets based on the CPTs to solve the dam surface deformation before and after aberrant interruptions. Finally, a deformation monitoring experiment was performed on an actual large reservoir dam. The effectiveness and accuracy of the abovementioned method were verified by comparing the results with measurements by a reversed pendulum monitoring system.

## 1. Introduction

Ground-based synthetic aperture radar interferometry (GB-InSAR) is a ground active microwave remote sensing technology that has been developed over the past decade. A ground-based synthetic aperture radar (GB-SAR) sensor can acquire two-dimensional (2D) radar images in the target area and measure the deformation component of each pixel of the line of sight (LOS). To date, this technology has been widely applied in the monitoring and analysis of various types of natural hazards, such as volcanoes, landslides, rockfall, and glaciers [1,2,3,4,5,6,7,8,9]. GB-InSAR application can also be extended to man-made structures, such as harbor infrastructures [10], dams [11] and bridges [12], providing a basis for structural safety analysis.

Reservoir dams are under the long-term influence of complex natural geological conditions and various external forces, such that the operating conditions of these dams are in constant change. A major reservoir dam structure often requires long-term stability monitoring as well as continuous safety monitoring during special periods, such as post-disaster periods, water storage, and flood discharge. Real-time processing methods for continuous image sequences as already reported in [13,14] can provide information on dam dynamics as well as the timely detection of defects and anomalies. Water storage and flood discharge affect the water level in the reservoir area and are directly related to dam deformation. GB-SAR can monitor dam deformation with extremely high spatial resolution, which is beneficial in analyzing the impact of different water conditions on the dam body. The dam deformation detection over a long time span with different water level conditions adopts the discontinuous monitoring mode of GB-SAR. The interferometric calculation in this mode generally needs to consider the repositioning errors of the GB-SAR rail [15], which is beyond the scope of this article.

Sparse discrete point monitoring is the main form of traditional geodetic deformation monitoring technology. The deformation is extracted by continuously collecting and comparing the position data of discrete points. Traditional geodetic methods, such as geometric leveling, total station, and GPS, usually have high single-point measurement accuracy. However, there are still serious deficiencies in the practical monitoring application of this technology. Key parts of the deformation body can be easily omitted when selecting the location of the monitoring points, such that the spatial integrity of the deformation information cannot be achieved. If a monitoring point is unstable or damaged, the monitoring data is prone to error or interruption, resulting in temporal deformation discontinuity. The deformation monitoring accuracy of the terrestrial laser scanner (TLS) depends on the accuracy of point cloud modeling and is relatively low in the absence of adequate feature points. This scanning technology is limited by distance measurement accuracy and cannot currently achieve ideal deformation monitoring accuracy. The accuracy of digital photogrammetry deformation monitoring technology also depends on the selection of feature points. As imaging occurs in the visible light band, it is difficult to carry out all-time deformation monitoring with digital photogrammetry. Compared with spaceborne InSAR technology, GB-SAR has a simple system architecture and a flexible parameter configuration, and in a continuous monitoring mode does not have a spatial baseline, obviating the consideration of the impact of the guide rail baseline error or the reference phase and topographic phase errors [13]. The imaging time of GB-SAR is generally short. For example, with the maximum detection range set to 1 km, it takes approximately five minutes for an IBIS-L system to acquire one view of GB-SAR images [16], whereas it takes as little as five seconds for the FastGBSAR system to do the same [17]. GB-SAR can continuously take images of the same area with an extremely high sampling frequency, flexibly determine the temporal baseline according to the deformation characteristics of the target, and carry out periodic deformation monitoring. GB-SAR can select observation stations for a dam structure and establish specific geometric scenes according to prior deformation information, where the theoretical accuracy of deformation detection can reach 0.1 mm [18].

Currently, available GB-SAR systems (e.g., IBIS-L, LISA, and FastGBSAR) use interferometric phase integration over time to calculate deformation values to maximally prevent the phase wrapping incurred with images obtained over a long time span [19,20,21]. On this basis, the GPRI system (GAMMA, Bern, Switzerland) applies the interferometric phase moving average to reduce the influence of noise and meteorological changes [2]. The abovementioned two methods for interferometric calculation are actually only applicable to the continuous monitoring mode of GB-SAR. One-dimensional (1D) phase unwrapping over time of a phase series obtained in the fast continuous monitoring mode can generally produce good results. However, in actual monitoring, various factors inside and outside the radar sensor interrupt smooth continuous deformation monitoring. For example, severe weather conditions, such as sudden rain and snow, lead to aberrant changes in the GB-SAR monitoring environment, which directly affect the stability and accuracy of the signal itself. As a result, the image data for some time periods cannot be used in the interferometric calculation. In addition, the operation of radar equipment may be interrupted due to faults, for example. The phases of the two views of the image before and after the restart of the operation cannot be directly connected, and possible phase wrapping over time leads to a missing phase cycle, thereby giving rise to aberrant interferometric calculation results. The atmospheric phase errors of the image series acquired by the GB-SAR continuous monitoring mode are generally corrected using reference points, the selection of which has a direct impact on the calculation results. Under the combined influences of atmospheric environment disturbances and noise, aberrant hopping of the observed phases at the reference points and monitoring points can directly affect the interferometric calculation values of adjacent areas. Such erroneous results continue to accumulate in subsequent calculations. Therefore, extremely stringent requirements are placed on the data quality and reliability of the reference points. Generally, the further away the reference points from the monitoring points, the higher the instability of the interferometric phase unwrapping, and the more likely there is the generation of aberrant results, leading to the mixing of the real phase with noise and anomalies, and the low reliability of the obtained deformation series. In order to analyze the impact of different water conditions on the dam, GB-SAR deformation monitoring needs to maintain reliability for a long time to provide a continuous deformation sequence. However, the aberrant interruption of the data cuts the temporal continuity of the deformation, which brings difficulties to the continuous deformation analysis of the dam.

In this study, differential GB-InSAR based on image subsets and coherent point targets (CPTs) was proposed to address the loss of the spatial or temporal reference in deformation calculations by traditional interferometric methods in continuous monitoring mode. During the continuous monitoring of the dam, even if the image collection is interrupted, this method can maintain the reference for deformation calculation with high accuracy, making it possible to analyze the impact of continuous changes in water conditions on the dam using GB-SAR. First, the observation time period for each image subset was determined. GB-SAR images were acquired in a continuous mode over time periods with relatively stable meteorological changes and used to form multiple image subsets. Then, a multi-threshold method was used to select CPTs from the continuous image data of each image subset. An average image for each image subset was then calculated. Finally, the CPTs were used to construct an irregular triangular network, the interferometric phase was solved by using the spatial adjacency relation of the CPTs, and an atmospheric phase correction was applied to extract the deformation of each CPT.

The rest of the paper is organized as follows. The multi-threshold method for CPT selection is described in Section 2. In Section 3, interferometry is applied to the average image of discrete image subsets. In Section 4, a reservoir dam deformation monitoring experiment is carried out to implement the technical process designed in this study, and observation data from a reversed pendulum monitoring system at the central axis of the dam are used for verification and analysis. The study is summarized in Section 5.

## 2. Multi-Threshold Strategy for Coherent Point Target Selection

To ensure reliability and accuracy, the deformation calculation is generally based on stable point targets, i.e., CPTs. Compared with other pixels, the intensity and phase value of CPTs can maintain higher stability under the influence of atmosphere and noise during continuous observation. Spaceborne SAR looks down on the ground from a high altitude, while GB-SAR looks sideways at the target area. The GB-SAR beam angle is wider than spaceborne SAR, forming a special fan-shaped grid coordinate system. When the surface topography changes are complicated, the shadow area may be generated during GB-SAR imaging. Before using GB-SAR to acquire images, it is usually necessary to set the longest observation distance, also known as the cutoff distance. Within this distance, the microwave signal may not meet any target in some directions to form a reflected signal. In these two cases, the corresponding GB-SAR image pixels still have observation values, which are collectively referred to as the false signals of GB-SAR in this paper. The unwrapped phase series of the time series of the false signals may also have a relatively low amplitude deviation index (ADI). The coherence threshold [22,23] considers the strong scattering signal of the CPT but neglects the stability of the CPT, whereas the ADI [24] and the phase stability analysis method only take into account the CPT stability while neglecting the strong scattering signal. Current InSAR time series analysis based on discrete stable point targets combines a variety of threshold methods to reliably extract more CPTs. Therefore, greater flexibility is required for GB-SAR CPT selection. We designed the following multi-threshold strategy to extract CPTs from a continuous series of GB-SAR images.

### 2.1. Extraction of Candidate Points Using Double Thresholds of Average TSNR and Average Coherence

The thermal signal-to-noise ratio (TSNR) [25] of the GB-SAR pixel can be visually inspected to determine the relative energy intensity. In general, the higher the TSNR, the more accurate the observed phase. To remove most of the background false signals, we first calculated the average TSNR of the image series using the following formula:

(1) $$TSNR_{ave}=\frac{1}{K}\sum_{k=1}^{K}TSNR_{k}$$

where *TSNR_k_* is the TSNR of the pixel in the *k*th GB-SAR image, *TSNR_ave_* is the average TSNR of the pixel TSNR series, *K* is the total number of continuous images in the image series, and *k* is the image serial number.

The TSNR value of a GB-SAR pixel was related to the distance of the pixel from the center of the synthetic aperture. The number and distribution of false signals in GB-SAR images were clearly different in different monitoring scenarios. Therefore, an appropriate threshold could be determined by analyzing the effect of removing false signals.

The average coherence of the image series was calculated using Formula (2). The coherence distribution was analyzed after the average TSNR and a reasonable threshold is set to select candidate point targets:

(2) $$\gamma_{ave}=\frac{1}{K-1}\sum_{k=1}^{K-1}\gamma_{k}$$

where *γ_k_* is the coherence of the pixel in the *k*th GB-SAR image with respect to the first image and *γ_ave_* is the average coherence of the pixel series.

Because the image series have relatively short time spans, the average TSNR and the coherence threshold could be used to remove most of the false signals, weak signals, and unstable signals.

### 2.2. Removal of Low-Quality GB-SAR Images

Aberrant interferences in the imaging process result in low quality GB-SAR images. Images with clearly compromised quality should be detected and eliminated after the candidate point target selection. Due to the influence of the atmospheric environment, after selecting the first image as the reference image for coherence calculation, the coherence sequence may have an obvious trend term. In order to eliminate the images with significantly poor quality, we used a simple linear fitting to extract the linear trend term of the coherence series of each pixel, and calculated the residual value to the linear trend. The following Formula (3) was used to calculate the absolute coherence residual Δ*γ_residual_* to analyze the coherence variation of the point target.

(3) $$\Delta \gamma_{residual}=\left|\gamma_{k}-\gamma_{linear}\right|$$

where Δ*γ_residual_* is the absolute coherence residual relative to the linear fitting value.

The distribution of the absolute coherence residual of the candidate point targets in each view of the image was analyzed. A decorrelation threshold was set to calculate the ratio of the number of severely decorrelated point targets (typically Δ*γ_residual_* > 0.1) to the total number of candidate point targets. This ratio was an indicator of the overall quality of the GB-SAR images and was used to remove excessively poor-quality images.

### 2.3. ADI Threshold Method for CPT Screening

Amplitude values are generally not sensitive to atmospheric effects. After the removal of low-quality GB-SAR images, the stability of the target pixel was ensured by using the ADI threshold method to further analyze and screen candidate point targets. The ADI was calculated using Formula (4) [24]:

(4) $$D_{A}=\frac{\sigma_{A}}{m_{A}}$$

where *σ_A_* and *m_A_* are the standard deviation and mean value of the pixel amplitude, respectively, in the GB-SAR image sequence.

When the ADI threshold is strictly set, false signals can be removed relatively completely, and the number of extracted point targets will significantly decrease at the same time. In practical processing, the ADI threshold value should be determined flexibly according to the quality of the observed images. The setting of ADI threshold should also ensure a sufficient CPT density to facilitate the deformation calculation in which the CPTs are used to form an irregular triangulation network. Therefore, the threshold was determined by a synthetic statistical analysis [24,26]. Because GB-SAR images are not subjected to radiometric calibration, the original signal strength also varies to some extent under a changing ambient atmosphere. The ADI should be calculated after removing the trend term from the strength series of each point target.

## 3. Differential GB-InSAR Based on Image Subsets

**Figure 1:**
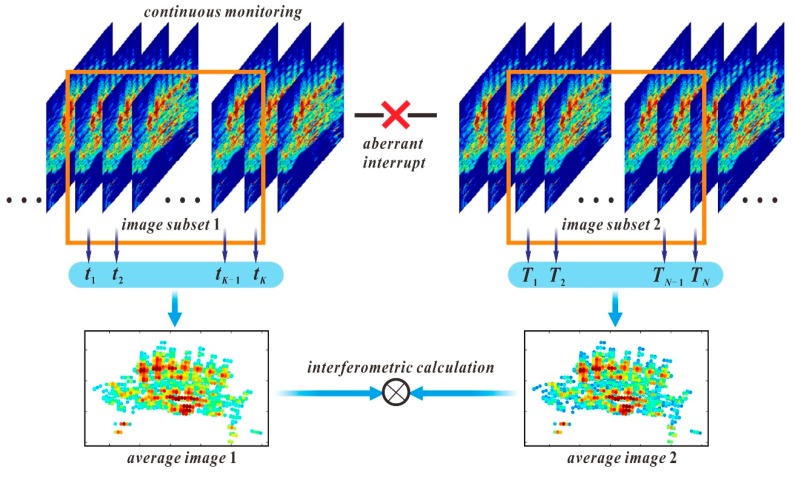
Conceptual framework of differential ground-based synthetic aperture radar interferometry (GB-InSAR) calculation using image subsets.

In this section, a differential GB-InSAR analysis method based on the average images of image subsets is proposed. The conceptual framework of this calculation method is shown in Figure 1. For aberrant interruptions of continuous image acquisition, the phase must be spatially unwrapped to prevent the loss of the phase cycle as in the traditional method. However, obvious interference factors, such as atmospheric effects and random noise in single GB-SAR images make it difficult to obtain ideal accuracy in a DInSAR calculation by using only two images before and after the interruption. The advantage offered by GB-SAR in high-frequency image sampling was reduction of the impact of atmospheric environmental variations. A certain number of images were continuously acquired in each period as an independent image subset, within which the multi-threshold method was used to select the CPTs in the layers. The CPTs are used to calculate the average image of the image subset and in the differential interferometric processing of the average images.

### 3.1. Calculation of Average Image of an Image Subset

**Figure 2:**
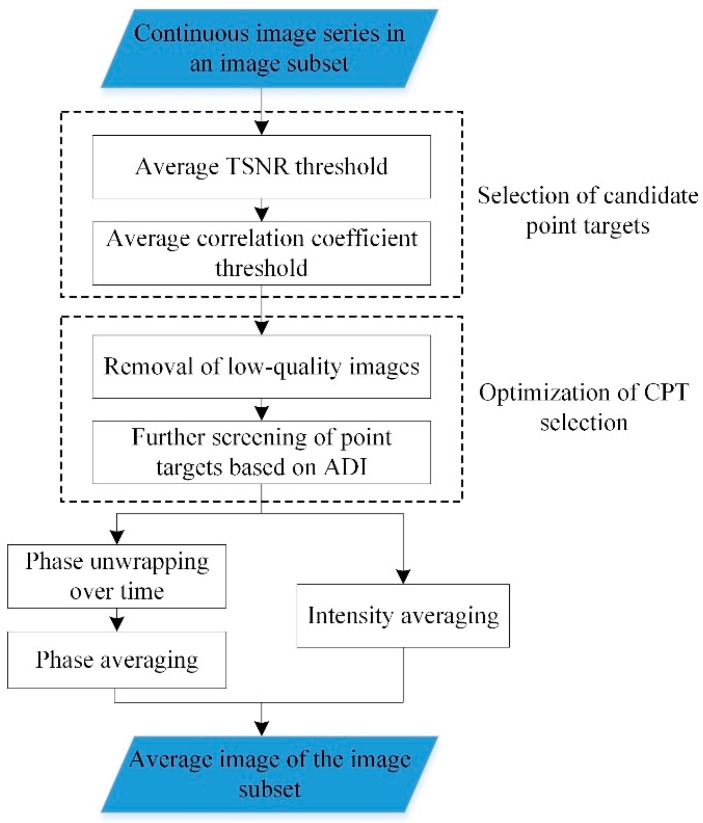
Processing of a continuous image series in an image subset and calculation of the average image of the image subset. Abbreviations: TSNR, thermal signal-to-noise ratio; ADI, amplitude deviation index; CPT, coherent point targets.

The discontinuous deformation estimation method using GB-SAR image subsets was based on the concept of spaceborne DInSAR technology and fully utilized the high sampling rate feature of the GB-SAR system. In monitoring ground surface micro-deformation, the ground surface is usually considered not to deform over a short period of time (e.g., a few hours). The observed phase and signal strength of the continuous images in the image subset usually have high quality and stability. Averaging the complex vector signal can mitigate the impacts of both the complex vector signal and other additive noise [27]. The basic steps for processing a continuous image series of an image subset are shown in Figure 2.

Phase unwrapping was required prior to the averaging of the observed phase series within the image subset. First, the first image of the image subset was usually selected as the master image and the other views were treated as slave images. If the phase variation is relatively slow, 1D phase unwrapping over time can be directly performed for each candidate point without 2D spatial phase unwrapping. The phase unwrapping was processed point-by-point, and the adjacent phase difference was calculated to remove isolated point targets with anomalous deformations. Then, the average phase of each candidate point target was calculated using Formula (5) [27]:

(5) $$\varphi =\frac{1}{K}\sum_{k=1}^{K}\left[∠\left(f_{1}\right)+∠\left(W^{-1}\left(f_{1}\cdot conj\left(f_{k}\right)\right)\right)\right]$$

where *K* is the total number of GB-SAR images in the image subset; *k* is the image serial number; *f*_1_ and *f_k_* are the first and the *k*th complex data of the same pixel in the image subset; *conj* (·) denotes taking the conjugate of a complex number; *W*^−1^ (·) is the phase unwrapping operator; and ∠ (·) is the phase extraction operator.

Finally, the average phase and the average signal strength were combined to fuse the image subset into a one-view average complex image, and differential interferometric processing was subsequently carried out using the average image of each image subset.

### 3.2. Differential GB-SAR Interferometry Model Using Averaged Image of Image Subset

An interferometric phase model was used to calculate the deformation in differential GB-InSAR. For spaceborne DInSAR technology, the main values of the observed phase included the flat-earth trend phase, the topographic phase, the deformation phase, the atmospheric disturbance phase, and the noise phase. GB-SAR is a simpler system. In continuous monitoring mode, GB-SAR has a fixed rail, no spatial baseline, and the repeated installation error of the rail is zero. The differential interferometric model based on the average image of the GB-SAR image subset can be simplified as follows:

(6) $$\varphi_{diff}=\varphi_{def}+\varphi_{atm}=-\frac{4\pi }{\lambda }\cdot d+\varphi_{atm}+\varphi_{noi}$$

where $\varphi_{diff}$ is the interferometric phase, $\varphi_{def}$ is the deformation phase, $\varphi_{atm}$ is the atmospheric phase, $\varphi_{noi}$ is the noise, *d* is the deformation value, and *λ* is the microwave wavelength.

After the differential interferometric calculation based on the average image of the image subset was completed, the spatial correlation of the observed phases of the point targets was considered by using the CPTs to construct an irregular triangular network. A functional model for the network adjustment was established by connecting the baselines at the network edges to form CPT pairs, and the deformation was estimated by parameter adjustment. The relative deformation phase of a point pair $\Delta \varphi_{diff}^{i,j}$ after noise reduction is as follows:

(7) $$\Delta \varphi_{diff}^{i,j}=\varphi_{def}^{i}-\varphi_{def}^{j}+\Delta \varphi_{atm}^{i,j}$$

where *i* and *j* are the ID numbers of the CPT pairs after the construction of the network, $\varphi_{def}^{i}$ is the deformation phase of CPT *i*, $\varphi_{def}^{j}$ is the deformation phase of CPT *j*, and $\Delta \varphi_{atm}^{i,j}$ is the differential atmospheric phase of the point pair *ij* at the network edge.

The initial calculation data were required to obtain the absolute deformation. Generally, prior information of the measurement area can be combined to identify a relatively stable area in the optimal observation field of GB-SAR. Then, one or more stable point targets are selected in the stable area as reference points with zero deformation and used to calculate the deformation and atmospheric phase at other point targets.

### 3.3. Atmospheric Correction Using Irregular Triangular Network

The atmospheric phase is corrected by selecting one or more reference points in the stable area near the monitoring area. Because the reference point does not deform over the entire monitoring process, the actual measured phase of the reference point can be regarded as resulting from atmospheric variations during the monitoring period. Considering the relatively small horizontal and vertical spans of the dam area in a GB-SAR image, the local atmospheric consistency is relatively good along the propagation path. Using the reference point as the datum, the spatially relevant component of the atmospheric phase can be generally corrected satisfactorily by using a univariate linear model [16].

**Figure 3:**
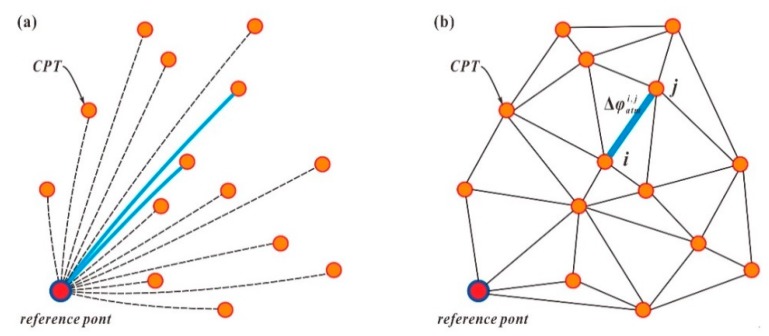
Atmospheric correction methods for continuous monitoring: (**a**) traditional atmospheric correction method for continuous monitoring, which is easily affected by the long distance between the monitoring points and the reference point; and (**b**) atmospheric correction method based on an irregular triangular network.

In the traditional atmospheric correction method, the interference between the monitoring points and the reference points is calculated directly, as shown in Figure 3a. The stability and accuracy of the results are often affected by the long distance between the monitoring points and the reference point. The correction method we adopted is based on constructing an irregular triangular network of CPTs. As shown in Figure 3b, the atmospheric correction of the edge difference phase was performed. After an interferometric calculation was carried out for each average image using the master average image, the atmospheric phase at CPT *i* was as follows:

(8) $$\varphi_{atm}^{i}=a\cdot r_{i}+b$$

where *a* and *b* are a fixed coefficient and a correction, respectively, that can be solved using at least two reference points; and *r_i_* is the distance from the CPT to the center of the synthetic aperture. The differential atmospheric phase of the CPT pair *ij* at the network edge is as follows:

(9) $$\Delta \varphi_{atm}^{i,j}=a\cdot dr_{i,j}$$

where *dr_i_*,*_j_* is the difference in the distance from the CPT pair *ij* to the center of the synthetic aperture.

### 3.4. Deformation Calculation Using Weighted Least Squares Method

The differential atmospheric phase $\Delta \varphi_{atm}^{i,j}$ is deducted from the differential interferometric phase $\Delta \varphi_{diff}^{i,j}$ at the network edge *ij*, and the phase value is converted to the deformation value. The following observation equations were formulated:

(10) $${\hat{d}}_{i}-{\hat{d}}_{j}=\Delta d_{i,j}-v_{d}\;\left(i\neq j,\forall i,j=1,2,3,\cdots ,T\right)$$

where ${\hat{d}}_{i}$ is the estimated deformation of CPT *i*; Δ*d_i_*_,*j*_ is the relative deformation of the CPT pair *ij*; *v_d_* is the relative deformation residual of the CPT pair *ij*; and *T* is the total number of CPTs for which the connection relation is established.

A similar observation equation can be formulated for each baseline in the CPT network. Assuming there are Q baselines in the network, the observation equations for all of the baselines formed the following system:

(11) $$\underset{Q\times 1}{L}=\underset{Q\times T}{B}\cdot \underset{T\times 1}{X}+\underset{Q\times 1}{R}$$

(12) $$X={\left[{\hat{d}}_{1},{\hat{d}}_{2},{\hat{d}}_{3},\cdots ,{\hat{d}}_{T}\right]}^{T}$$

(13) $$B=\left[\begin{array}{lllll} 1 & 0 & \cdots & \cdots & 0\\ 0 & 1 & \cdots & \cdots & 0\\ \vdots & \vdots & \vdots & \vdots & \vdots \\ -1 & 1 & 0 & \cdots & 0\\ \vdots & \vdots & \vdots & \ddots & \vdots \end{array}\right]$$

where *B* is a coefficient matrix composed of 1 and −1 values; *L* is the vector of observed values, i.e., the relative deformations of the CPT pairs calculated using the original interferometric phases; *R* is a residual vector; and *X* is the CPT deformation vector to be determined.

The weighted least squares solution of *X* was calculated using parameter adjustment, and the deformation at each point target was obtained as follows:

(14) $$X={\left(B^{T}PB\right)}^{-1}B^{T}PL$$

The prior weight *P* of the CPT network edges can generally be taken as the differential phase stability factor *ps_i_*_,*j*_, which was calculated using Formula (15):

(15) $$ps_{i,j}=\frac{1}{\sqrt{\frac{\sum_{k=1}^{K}{\left(\Delta \varphi_{i,j}^{k}-\Delta {\bar{\varphi }}_{i,j}\right)}^{2}}{K}}}$$

where *K* is the total number of GB-SAR images in the image subset, *k* is the image serial number, $\Delta \varphi_{i,j}^{k}$ is the *k*th interferometric phase at the network edge *ij* of the CPT pair, and $\Delta {\bar{\varphi }}_{i,j}$ is the average interference phase.

## 4. Experimental Analysis

### 4.1. Experiment Overview

**Figure 4:**
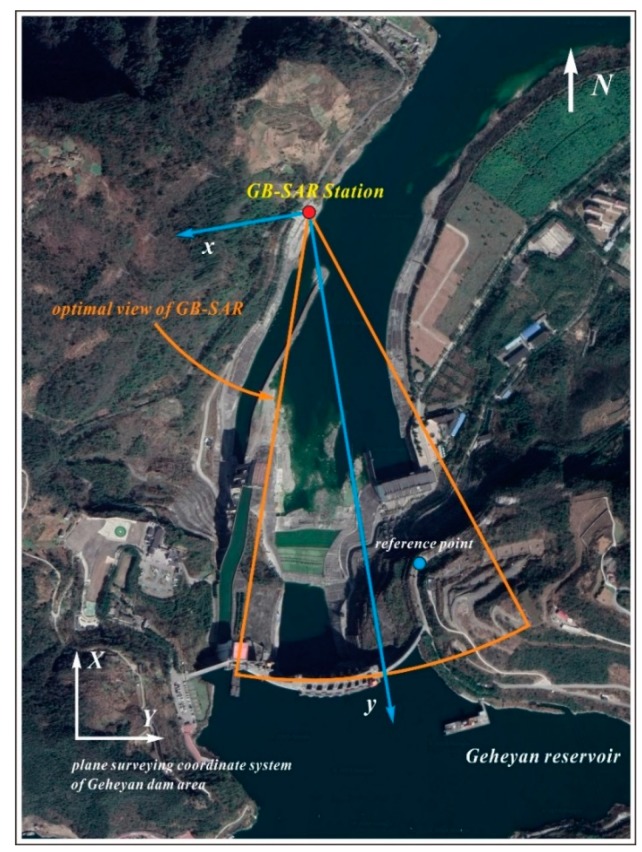
Ground-based synthetic aperture radar (GB-SAR) monitoring range in dam area.

The Geheyan water conservancy project consists of a concrete gravity arch dam, a release structure, a diversion-type hydropower station on the right bank, and a vertical ship lift on the left bank. In the experiment, an advanced GB-SAR system, the IBIS-L, was used to monitor the deformation of the Geheyan concrete gravity arch dam in the Qingjiang River and the adjacent area. Figure 4 shows the IBIS-L system that was installed on the left bank downstream of the dam, which is approximately 1 km away from the dam where the central line of sight of the radar is oriented towards the right side of the dam. Both the dam and the high slope on the right bank were within the optimal radar field of view, ensuring a good echo effect in the main monitoring area.

**Figure 5:**
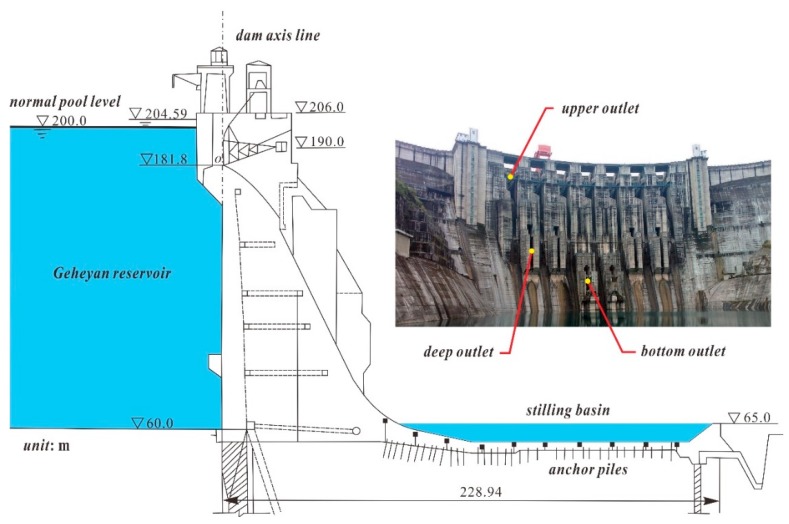
Side and front views of main Geheyan dam structure.

The main structure of body and surface of the Geheyan dam is shown in Figure 5. The Geheyan dam has a main dam crest elevation of 206 m, a total crest length of 665.45 m, and a maximum dam height of 151 m. Gravity dam sections are located on both banks. A composite concrete gravity dam was built over the riverbed, where the upper part (at an elevation above 150 m) is a gravity dam, and the lower part (at an elevation below 150 m) is an arch dam with an outer arc radius of 312 m. The dam has a total of seven upper outlets, four deep outlets, and two bottom outlets for dam emptying that also serve as diversion areas. The upper outlets have a weir top elevation of 181.8 m and are 12 m × 18.2 m in size. The deep outlets have an outlet bottom elevation of 134 m and are 4.5 m × 6.5 m in size. The bottom outlets have an outlet bottom elevation of 95 m and are 4.5 m × 6.5 m in size. All of the outlets are controlled by arc gates.

The algorithm proposed in this paper was directly verified using a monitoring experiment in the GB-SAR continuous mode to acquire image data. Image subsets were selected from the continuous image series at different time periods for analysis. The experiment began at 20:24 on 27 July 2013 and ended at 11:06 on 2 August 2013. A total of 1330 GB-SAR images were acquired. The basic parameters for data acquisition by the IBIS-L system are shown in Table 1.

**Figure 6:**
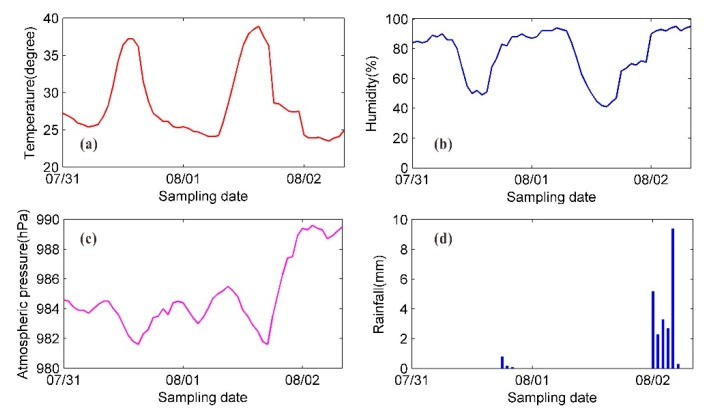
Variations in meteorological parameters during continuous deformation monitoring experiment: (**a**) temperature; (**b**) humidity; (**c**) atmospheric pressure; and (**d**) rainfall.

During the radar monitoring period, we used the weather monitoring station on the right bank slope of the dam to collect the meteorological parameters, i.e., the air temperature, the humidity, the atmospheric pressure, and the rainfall. Figure 6 shows the variation trends for the meteorological parameters. The temperature variation was relatively intense around noon and peaked at approximately 12:00, when the corresponding humidity was low.

### 4.2. Image Subset Selection and Average Image Calculation

The accuracy of the SAR deformation monitoring was evaluated from 00:00 on 27 July 2013 to the completion of the GB-SAR monitoring by simultaneously conducting reversed pendulum monitoring of the horizontal displacement at five elevation positions along the central axis of the Geheyan dam at 10-min sampling intervals. A total of 545 views of GB-SAR images were obtained that were coincident with the reversed pendulum observations.

**Figure 7:**
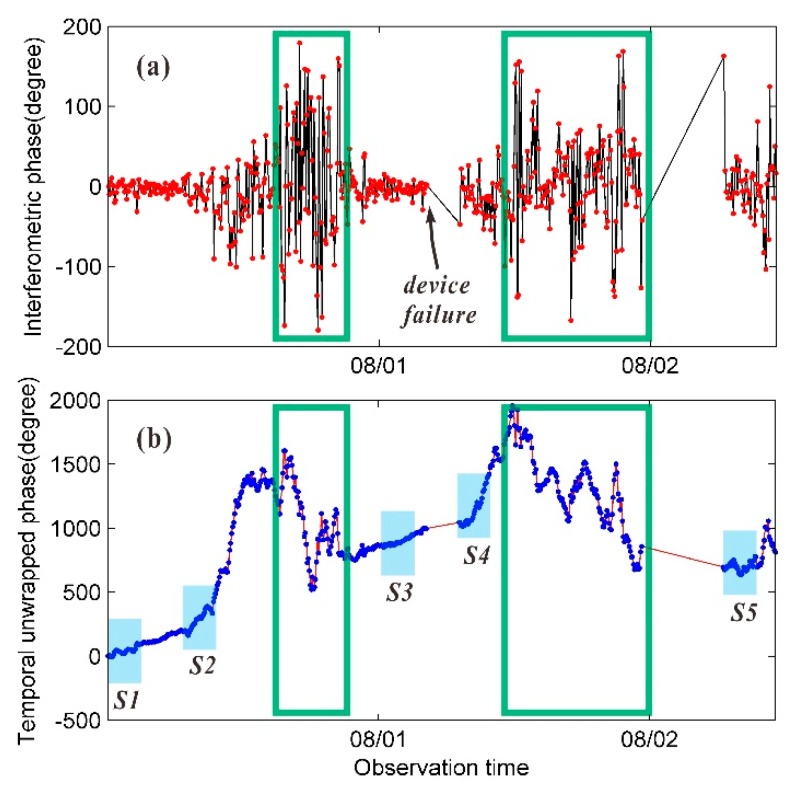
Interferometric phase series and unwrapped phase series of a high-TSNR pixel in the Geheyan dam area: (**a**) interferometric phase series of adjacent images; and (**b**) unwrapped phase series, where S1, S2, S3, S4, and S5 are the five selected image subset periods.

The temperature and humidity variation trends in Figure 6 show that the meteorological parameters varied relatively smoothly during the time periods before and after 00:00 and varied turbulently from 08:00 to 12:00. Similar patterns were observed for the continuous interferometric phase and the unwrapped phase of high-TNSR pixels. As shown in Figure 7, when the meteorological parameters varied smoothly, there was no observable decorrelation effect of the image series, and the observed phase values were highly stable. There were two significant rainfall events before the early morning of August 1 and 2 that significantly impacted the GB-SAR image quality. The interferometric phase between adjacent images fluctuates dramatically in the green-boxed area in Figure 7a, and the aberrations in the corresponding unwrapped phase in the green-boxed area in Figure 7b do not conform to the actual deformation pattern. In addition, radar equipment failures occurred twice on August 1 and 2, and several hours of image data were missing.

To overcome the influence effects of severe atmospheric environmental changes during the experiment and ensure continuity in the deformation data, the differential interferometric method based on the image subsets obtained in the present study was adopted. First, the image subset series was determined according to the degree of stability of the interferometric phase variation. Detailed information on the five selected image subsets is provided in Table 2.

The unwrapped phase series in Figure 7b reflects the atmospheric variation trend to some extent. The time periods in one day for image subsets S1 and S3 were similar, and time periods in one day for image subsets S2 and S4 were similar. The variation in the atmospheric environment of the measurement area was mild for the time periods of S1 and S3, and intense for the time periods of S2 and S4; however, a linear variation pattern can be observed in both cases. To facilitate a comparative analysis, the image subset S5, which exhibits notable nonlinear variations in the atmospheric environment, was also selected.

**Figure 8:**
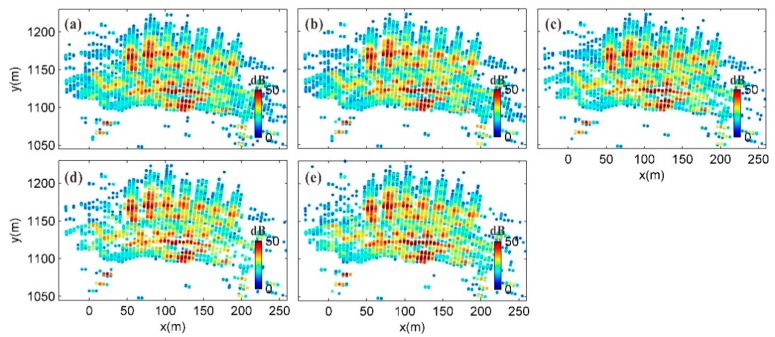
TSNR plots of average images of image subsets: (**a**–**e**) TSNR plots of average images of image subsets S1 to S5, respectively.

In each sub-image set, the double threshold method using the average TSNR and the coherence were first used to remove false signals and some weak signals as well as eliminate low-quality images. Then, the ADI threshold method was used to further remove false signals and screen for reliable CPTs. All of the thresholds were set flexibly based on the statistical results for the selected pixels. In image subset S1, the average TSNR threshold was set to 10 dB, the coherence threshold was set to 0.7, and the ADI threshold was set to 0.25 to produce 4892 point targets, accounting for 17.8% of the total number of pixels. To reduce the influence of meteorological variation for a long time series, 1D phase unwrapping was performed for the continuous image series in the image subset using the discrete CPTs. The average of the unwrapped phases was taken as the phase value of the average image, and the average TSNR of the CPTs in the image subset was used as the strength value of the average image. The same method was used to extract 4511, 4546, 3539, and 4080 CPTs from image subsets S2 to S5, respectively, and the corresponding average images were calculated. The TSNR plot of the average image corresponding to each image subset is shown in Figure 8.

### 4.3. Deformation Extraction by Interferometric Calculation

**Figure 9:**
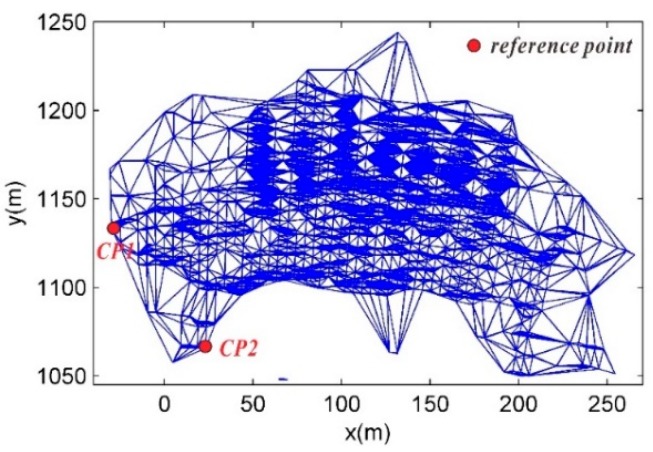
Irregular triangular network constructed using CPTs.

The deformation monitoring results for the last 10 years using traditional monitoring methods (the displacement meter and the total station) indicated that the high slopes on both sides of the Geheyan dam are stable, and the CPTs in this area can be selected as reference points. An irregular triangular network was constructed based on the CPTs of various interferograms, as shown in Figure 9. CP1 and CP2 are two CPTs at different elevations on the high slope of the right bank of the dam and were selected as reference points to calculate the parameters of the univariate atmospheric correction model. CP2 was a CPT at the bottom of the high slope of the right bank in the dam area and was selected as the reference point for the spatial phase unwrapping. In the unwrapping, atmospheric correction was first applied to the differential interferometric phases of the point pairs based on the difference in the distances between the point pairs on the network edges. Then, the reference point was taken as a zero-deformation point, and the reciprocal of the distance of each network edge was used as the weight in the weighted least squares calculation of the spatial phase unwrapping.

**Figure 10:**
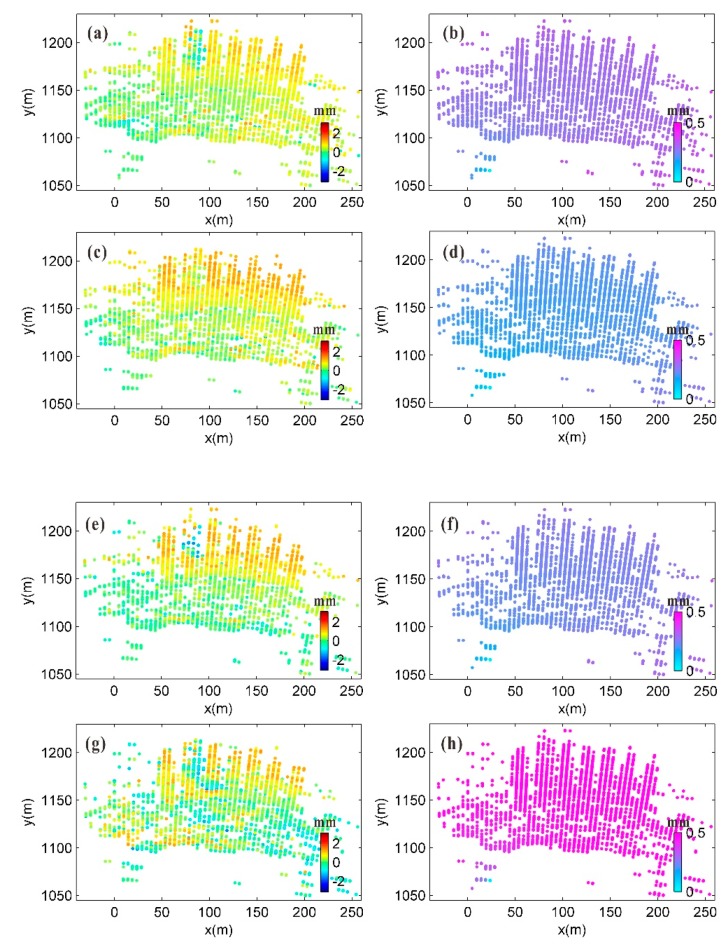
Cumulative deformation and corresponding GB-SAR calculation accuracy for each of four time periods: (**a**), (**c**), (**e**), and (**g**) show cumulative deformation for periods S1–S2, S1–S3, S1–S4, and S1–S5, respectively; (**b**), (**d**), (**f**) and (**h**) show estimated standard deviation in cumulative deformation for periods S1–S2, S1–S3, S1–S4, and S1–S5, respectively.

After performing the atmospheric correction and phase unwrapping, the phase result was calculated to yield the deformation. The deformation measurements and the estimated standard deviation of weighted least squares adjustment for each of the four time periods are shown in Figure 10.

**Figure 11:**
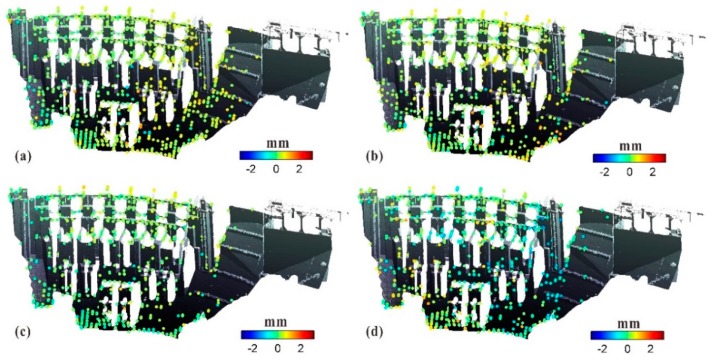
Mapping of cumulative deformation of dam surface to 3D dam model for four time periods: (**a**) S1–S2; (**b**) S1–S3; (**c**) S1–S4; and (**d**) S1–S5.

After the three-dimensional (3D) transformation of GB-SAR image coordinates assisted by TLS [28], the CPTs on the dam surface were directly extracted and mapped to the 3D dam model, as shown in Figure 11. This display of the deformation states of various parts of the dam structure enables a more intuitive analysis.

### 4.4. Analysis and Verification of Monitoring Results

**Figure 12:**
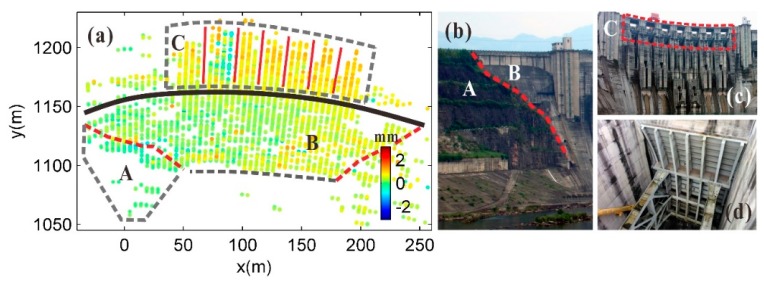
(**a**) Deformation distribution for time period of image subsets S1–S2; identification of deformation areas of the Geheyan dam structure in deformation distribution diagram: (**b**) boundary between right bank slope and dam structure, and (**c**) seven upper outlets of dam; (**d**) gate inside upper outlet release.

During the GB-SAR monitoring process, there was no notable water storage or flood discharge, and the surface deformation of the downstream dam structure was very small. In Figure 12, area A is the right bank slope of the dam, area B is the surface of the dam structure the main monitoring target, and area C contains the seven upper outlets at the dam crest. For the time period of image subset S1–S2, the CPTs associated with deformations below 0.5 mm in areas A and B accounted for nearly 80% of all of the CPTs. The deformation of the CPTs in the upper outlet channel area is larger than the deformation of the dam surface. We speculate that this difference may be due to considerable backscattering interference caused by the metal structure of the inner gate of the upper outlet, whereas the univariate linear atmospheric correction model effectively corrected the atmospheric phase of the dam surface but not that of the internal structural area of the upper outlet.

**Figure 13:**
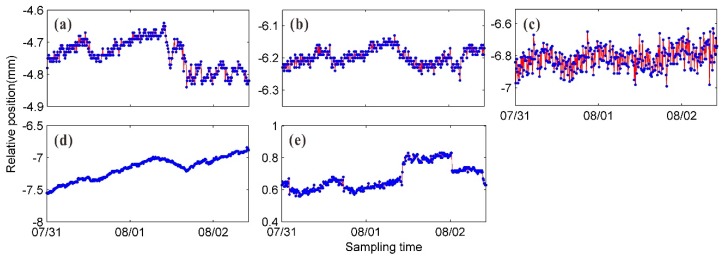
Deformation series at five elevation positions of dam central axis, as measured by reversed pendulum device: (**a**) 65 m (**b**) 105 m, (**c**) 122 m, (**d**) 145 m, and (**e**) 169 m.

The accuracy of the GB-SAR monitoring was further verified by identifying and extracting the CPTs at the central axis of the dam surface. The GB-SAR monitoring results were then compared with the reversed pendulum monitoring results at the central axis, which are shown in Figure 13. The cumulative deformations at elevations of 65, 105, 122, 145, and 169 m were automatically measured and recorded at 10-min sampling intervals by the reversed pendulum device.

**Figure 14:**
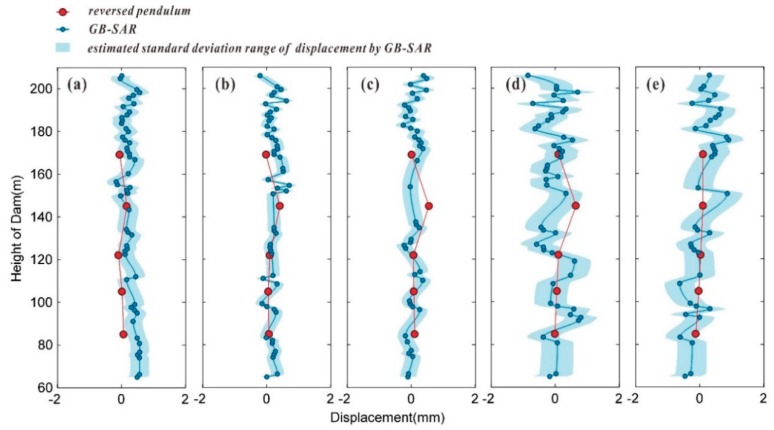
Comparison of monitoring results at central axis of dam surface by GB-SAR and reversed pendulum method for time intervals of (**a**) S1–S2, (**b**) S1–S3, (**c**) S1–S4, (**d**) S1–S5, and (**e**) S4–S5.

For both GB-SAR and the reversed pendulum device, the starting time was 7/31 0:00, and the cumulative deformations were calculated for the four time periods of image subsets S1–S2, S1–S3, S1–S4, and S1–S5. The deformation measured by both technologies was calculated in the upward radial direction of the dam. To retain the direct results of the GB-SAR method, the monitoring data at the central axis of the dam were not subjected to low-pass filtering or smoothing in the calculation, whereas segmental cubic polynomial interpolation was used to fit the deformation data. It can be clearly seen in Figure 14 that the impact of noise and atmospheric correction residuals always produce some fluctuations in the deformation of the central axis, as determined using GB-SAR. The fitted GB-SAR deformation curve was used to calculate the deformations at the five elevations along the central axis of the dam surface at which the reversed pendulum measurements were taken.

The cumulative deformations at the five elevations for the four time periods, as determined using the two aforementioned methods are given in Table 3. The differences between the cumulative deformations obtained by the two methods at the five elevations were very small, i.e., within 0.3 mm, except at three data points. The GB-InSAR results for S1–S2, S1–S3, and S1–S4 over a maximum time span of 31 h and were in good agreement with those obtained using the reversed pendulum method.

At 145 m elevation for the time period of image subsets S1–S4, the selected CPTs were sparse, and a relatively large difference was obtained between the results calculated using the two methods. In the time period of S1–S5, the maximum and minimum values measured by GB-SAR differed by 2.16 mm. The monitoring results at the five elevations obtained using the two methods were close to each other, but the overall deformation trend at the central axis was not consistent with the true deformation pattern of the dam. During data processing, the atmospheric phase for image subsets S1–S4 exhibited a notable linear trend, whereas that of the image subset S5 showed clear nonlinear fluctuations. The averaging method could not suitably weaken the impacts of noise and inhomogeneous meteorological disturbances for image subset S5. When the univariate linear model was used for the atmospheric correction of the interferogram of the S1–S5 average images, the effect of the correction was not significant. The presence of noise and inhomogeneous meteorological disturbances in the average image also increase the difficulty in phase unwrapping, resulting in a large estimated standard deviation in the phase unwrapping calculation results. The GB-SAR monitoring results for the time period of S1–S5 fluctuated more dramatically than those in the first three time periods.

The time span between image subsets S1 and S3 and between S4 and S5 was basically the same at approximately 24 h. We also used the differential GB-InSAR method to calculate the accumulated deformation for the S4–S5 period, as shown in Figure 14e. The difference between these results and those obtained using the reversed pendulum method is given in Table 3. It can be clearly seen that the fluctuation in the S4–S5 result obtained using GB-InSAR is much more severe than that for S1–S3. The difference between the results obtained by the two methods was relatively large, which also demonstrates that the volatility of S1–S5 results was not caused by a long time span. In addition, comparing the results for S1–S5 and S4–S5 shows that the dam surface deformation at the central axis had a high consistency. Thus, the interference calculation for S5 introduced a large error for the average image and the atmospheric correction. Therefore, when the image subset method is used for deformation detection, continuous images with smooth meteorological variations or notably linear trends should be selected for the image subset.

**Figure 15:**
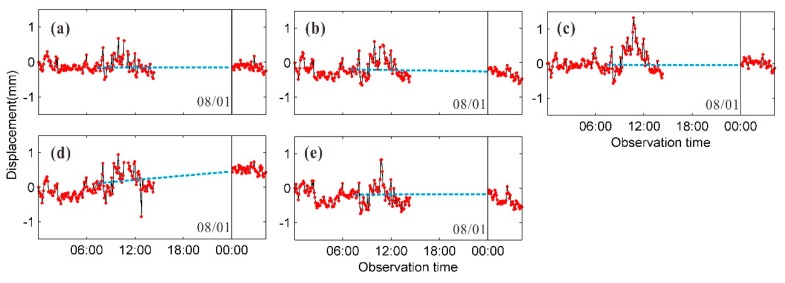
Connection of continuous monitoring data at five elevation positions of dam central axis using differential GB-InSAR results: (**a**) 65 m (**b**) 105 m, (**c**) 122 m, (**d**) 145 m, and (**e**) 169 m.

During the continuous deformation monitoring on 7/31 and 8/1, the quality of GB-SAR images from 15:00 to 24:00 was poor due to the impact of rainfall, and the reliability of the results calculated by the interferometric phase integration over time was low. We used the differential GB-InSAR method to restore the deformation calculation datum after the rain-affected period. Taking the five reversed pendulum measuring positions as an example, in order to reduce the noise influence, several CPTs adjacent to each position were selected for average calculation, and the GB-InSAR continuous deformation sequences were obtained before 7/31 15:00 and after 8/1 0:00. Deformation values at the five elevation positions were extracted from the S2–S3 difference GB-InSAR results, which were used to connect the deformation sequences before and after the rain-affected period. As shown in Figure 15, the connected data eliminates the adverse effects caused by the aberrant environment. And the observed dam surface deformation is generally consistent with the reversed pendulum monitoring sequences.

## 5. Conclusions

In this study, we used GB-SAR sensors to monitor the surface deformation of a large reservoir dam structure. This type of monitoring places high demands on the continuous, high-precision deformation monitoring by GB-InSAR technology. To solve the problem that the spatiotemporal datum for the deformation calculation is lost using traditional interferometric methods in the continuous monitoring mode, we implemented differential interferometry for the average images of the GB-SAR image subsets using CPTs that were selected by a multi-threshold method. To verify the effectiveness of the method proposed in this paper, we experimentally monitored the deformation of an actual large, complex reservoir dam. In the experiment, differential GB-InSAR based on image subsets was used to calculate the cumulative deformation of the dam for multiple time periods. A 3D transformation technology of the image coordinates was used to extract the deformation values at the central axis of the dam surface from the differential interferometry results. These deformation values were then compared with those obtained using a reversed pendulum monitoring system.

Discontinuous processing is used in the differential GB-InSAR based on image subsets, and high-quality average images are required for differential interferometry. Thus, the obtained deformation values can achieve calculation accuracy at the sub-millimeter level over a relatively short time span. Continuous images with stable meteorological variations or clear linear trends should be used as image subsets to ensure the accuracy of the results. The proposed method can well resolve the problem of spatiotemporal datum loss in the application of GB-SAR to continuous deformation monitoring and can maintain the spatiotemporal continuity of the deformation monitoring results. Differential GB-InSAR technology based on image subsets can be applied to areal deformation monitoring and prediction, and to early warning systems for dams, supported slopes, rock slopes, and landslides that have high requirements for continuous, real-time or dynamical data.

## Figures and Tables

**Table 1:** Basic equipment information for GB-SAR for deformation monitoring experiment.

**Antenna Type**	Gain	20 dBi
	Polarization mode	VV
**Signal Type**	Frequency band and wavelength	Ku/1.78 cm
	Bandwidth	300 M (17.05–17.35 GHz)
**Synthetic Aperture Length**		2 m
**Resolution**	Range	5 m
	Azimuth	4.4 mrad
**Maximum Monitoring Distance Setting**		1300 m
**Average Image Acquisition Duration**		5.3833 min

**Table 2:** Selection of continuous images in image subsets.

Image Subset No.	Starting Image No.	Acquisition Time M/D h:m	Last Image No.	Acquisition Time M/D h:m	Number of Images
S1	1	7/31 00:01	30	7/31 02:37	30
S2	78	7/31 07:01	107	7/31 09:38	30
S3	267	8/01 00:04	296	8/01 02:40	30
S4	314	8/01 07:08	343	8/01 09:46	30
S5	494	8/02 06:31	523	8/02 09:09	30

**Table 3:** Difference between monitoring results at central axis of dam surface obtained by GB-SAR method and reversed pendulum method.

Elevation/m	85			105			122			145			169		
Time Interval	P	S	Δ	P	S	Δ	P	S	Δ	P	S	Δ	P	S	Δ
**S1–S2**	0.07	0.46	0.39	0.02	0.29	0.27	−0.09	0.13	0.22	0.17	0.20	0.03	−0.05	0.25	0.30
**S1–S3**	0.07	0.01	−0.06	0.05	0.18	0.13	0.10	0.16	0.06	0.41	0.21	−0.20	−0.02	0.24	0.26
**S1–S4**	0.11	−0.18	−0.29	0.08	0.13	0.05	0.07	−0.12	−0.19	0.55	0.04	−0.51	0.01	0.29	0.28
**S1–S5**	−0.01	−0.27	−0.26	0.05	−0.11	−0.16	0.10	0.12	0.02	0.65	0.11	−0.54	0.11	0.18	0.07
**S4–S5**	−0.12	−0.56	−0.44	−0.03	−0.57	−0.54	0.03	−0.03	−0.06	0.10	0.56	−0.46	0.10	0.46	−0.36

Note: P denotes reversed pendulum monitoring method, S denotes GB-SAR monitoring method, and Δ denotes the difference between the P and S results.
